# Supplementary material for: Modeling glioblastoma heterogeneity as a dynamic network of cell states
Source: Mol Syst Biol. 2021 Sep 16;17(9):e10105. doi: 10.15252/msb.202010105 (PMC8444284; doi:10.15252/msb.202010105)
Supplement: Supplementary file 5 — Source Data for Figure 3 [file MSB-17-e10105-s001.zip › Figure3A_sourcedata/GSEA_3065/nosigdb_state5.GseaPreranked.1622718435914/LARSSON_STATE_4.html]

Details for gene set LARSSON\_STATE\_4[GSEA]

|  || Dataset | state5 |
| Phenotype | NoPhenotypeAvailable |
| Upregulated in class | na\_neg |
| GeneSet | LARSSON\_STATE\_4 |
| Enrichment Score (ES) | -0.68518084 |
| Normalized Enrichment Score (NES) | -2.3912947 |
| Nominal p-value | 0.0 |
| FDR q-value | 0.0 |
| FWER p-Value | 0.0 |
Table: GSEA Results Summary

  

Fig 1: Enrichment plot: LARSSON\_STATE\_4      
 Profile of the Running ES Score & Positions of GeneSet Members on the Rank Ordered List

  

| PROBE | GENE SYMBOL | GENE\_TITLE | RANK IN GENE LIST | RANK METRIC SCORE | RUNNING ES | CORE ENRICHMENT || 1 | BRCA1 |  |  | 522 | 0.305 | -0.0471 | No |
| 2 | ORC6 |  |  | 806 | 0.240 | -0.0698 | No |
| 3 | CENPF |  |  | 1531 | 0.149 | -0.1456 | No |
| 4 | ATAD2 |  |  | 2545 | 0.077 | -0.2567 | No |
| 5 | RAD51AP1 |  |  | 2905 | 0.057 | -0.2950 | No |
| 6 | SGO1 |  |  | 3060 | 0.049 | -0.3104 | No |
| 7 | NCAPG |  |  | 3194 | 0.041 | -0.3238 | No |
| 8 | SMC2 |  |  | 3374 | 0.032 | -0.3427 | No |
| 9 | FANCI |  |  | 3375 | 0.032 | -0.3415 | No |
| 10 | ESCO2 |  |  | 3672 | 0.021 | -0.3740 | No |
| 11 | GTSE1 |  |  | 3738 | 0.018 | -0.3806 | No |
| 12 | KIF15 |  |  | 3950 | 0.009 | -0.4040 | No |
| 13 | MCM4 |  |  | 4534 | -0.015 | -0.4691 | No |
| 14 | MCM10 |  |  | 4542 | -0.016 | -0.4692 | No |
| 15 | CDC45 |  |  | 4563 | -0.017 | -0.4709 | No |
| 16 | CENPK |  |  | 4675 | -0.021 | -0.4826 | No |
| 17 | SGO2 |  |  | 4709 | -0.022 | -0.4854 | No |
| 18 | CDK1 |  |  | 4766 | -0.025 | -0.4908 | No |
| 19 | EZH2 |  |  | 4858 | -0.028 | -0.5000 | No |
| 20 | MCM7 |  |  | 4986 | -0.032 | -0.5130 | No |
| 21 | GINS2 |  |  | 5024 | -0.034 | -0.5159 | No |
| 22 | CCNB1 |  |  | 5111 | -0.037 | -0.5242 | No |
| 23 | UHRF1 |  |  | 5337 | -0.046 | -0.5477 | No |
| 24 | NUSAP1 |  |  | 5389 | -0.048 | -0.5516 | No |
| 25 | CLSPN |  |  | 5614 | -0.058 | -0.5746 | No |
| 26 | TOP2A |  |  | 5722 | -0.063 | -0.5843 | No |
| 27 | KPNA2 |  |  | 5811 | -0.066 | -0.5917 | No |
| 28 | CHAF1A |  |  | 6157 | -0.082 | -0.6274 | No |
| 29 | MKI67 |  |  | 6211 | -0.086 | -0.6301 | No |
| 30 | RRM1 |  |  | 6298 | -0.090 | -0.6363 | No |
| 31 | RRM2 |  |  | 6308 | -0.091 | -0.6339 | No |
| 32 | CENPM |  |  | 6368 | -0.093 | -0.6369 | No |
| 33 | ASF1B |  |  | 6400 | -0.095 | -0.6368 | No |
| 34 | ZWINT |  |  | 6453 | -0.099 | -0.6389 | No |
| 35 | SMC4 |  |  | 6517 | -0.102 | -0.6421 | No |
| 36 | E2F1 |  |  | 6528 | -0.103 | -0.6392 | No |
| 37 | KIF4A |  |  | 6529 | -0.103 | -0.6353 | No |
| 38 | MCM5 |  |  | 6553 | -0.105 | -0.6339 | No |
| 39 | PIMREG |  |  | 6566 | -0.106 | -0.6312 | No |
| 40 | DBF4 |  |  | 6572 | -0.106 | -0.6277 | No |
| 41 | MAD2L1 |  |  | 6600 | -0.108 | -0.6266 | No |
| 42 | LMNB1 |  |  | 6638 | -0.110 | -0.6265 | No |
| 43 | FBXO5 |  |  | 6775 | -0.119 | -0.6373 | No |
| 44 | MND1 |  |  | 6972 | -0.135 | -0.6542 | No |
| 45 | PBK |  |  | 7010 | -0.138 | -0.6531 | No |
| 46 | GMNN |  |  | 7224 | -0.158 | -0.6711 | No |
| 47 | TMPO |  |  | 7296 | -0.167 | -0.6727 | No |
| 48 | CENPH |  |  | 7329 | -0.171 | -0.6698 | No |
| 49 | TK1 |  |  | 7377 | -0.178 | -0.6683 | No |
| 50 | CENPN |  |  | 7528 | -0.194 | -0.6778 | Yes |
| 51 | TPX2 |  |  | 7549 | -0.197 | -0.6725 | Yes |
| 52 | MCM3 |  |  | 7552 | -0.198 | -0.6652 | Yes |
| 53 | UBE2T |  |  | 7561 | -0.199 | -0.6585 | Yes |
| 54 | BIRC5 |  |  | 7596 | -0.204 | -0.6545 | Yes |
| 55 | PCLAF |  |  | 7734 | -0.223 | -0.6615 | Yes |
| 56 | CENPU |  |  | 7871 | -0.248 | -0.6673 | Yes |
| 57 | DHFR |  |  | 7878 | -0.250 | -0.6584 | Yes |
| 58 | H2AFX |  |  | 7965 | -0.269 | -0.6579 | Yes |
| 59 | CKS2 |  |  | 7972 | -0.270 | -0.6482 | Yes |
| 60 | HMGB2 |  |  | 7973 | -0.271 | -0.6379 | Yes |
| 61 | HIST1H1C |  |  | 7978 | -0.271 | -0.6280 | Yes |
| 62 | MYBL2 |  |  | 8041 | -0.283 | -0.6242 | Yes |
| 63 | TMSB15A |  |  | 8046 | -0.284 | -0.6138 | Yes |
| 64 | RFC2 |  |  | 8049 | -0.285 | -0.6032 | Yes |
| 65 | DTYMK |  |  | 8075 | -0.292 | -0.5948 | Yes |
| 66 | CDKN3 |  |  | 8079 | -0.293 | -0.5840 | Yes |
| 67 | PCNA |  |  | 8082 | -0.293 | -0.5730 | Yes |
| 68 | PHF19 |  |  | 8093 | -0.295 | -0.5629 | Yes |
| 69 | DUT |  |  | 8118 | -0.300 | -0.5541 | Yes |
| 70 | HIST1H1A |  |  | 8148 | -0.309 | -0.5456 | Yes |
| 71 | HIST1H1E |  |  | 8176 | -0.320 | -0.5365 | Yes |
| 72 | CKS1B |  |  | 8241 | -0.336 | -0.5308 | Yes |
| 73 | LSM5 |  |  | 8242 | -0.337 | -0.5180 | Yes |
| 74 | UBE2C |  |  | 8278 | -0.348 | -0.5087 | Yes |
| 75 | NUDT1 |  |  | 8359 | -0.373 | -0.5034 | Yes |
| 76 | HNRNPD |  |  | 8436 | -0.402 | -0.4967 | Yes |
| 77 | HIST1H1B |  |  | 8437 | -0.402 | -0.4813 | Yes |
| 78 | PTTG1 |  |  | 8446 | -0.406 | -0.4667 | Yes |
| 79 | NUCKS1 |  |  | 8502 | -0.435 | -0.4563 | Yes |
| 80 | HIST1H1D |  |  | 8520 | -0.443 | -0.4413 | Yes |
| 81 | ALYREF |  |  | 8535 | -0.449 | -0.4258 | Yes |
| 82 | SNRPD1 |  |  | 8537 | -0.451 | -0.4087 | Yes |
| 83 | RAN |  |  | 8555 | -0.463 | -0.3929 | Yes |
| 84 | RPA3 |  |  | 8572 | -0.471 | -0.3767 | Yes |
| 85 | TYMS |  |  | 8599 | -0.488 | -0.3611 | Yes |
| 86 | HMGN2 |  |  | 8608 | -0.496 | -0.3430 | Yes |
| 87 | HMGB1 |  |  | 8653 | -0.527 | -0.3279 | Yes |
| 88 | SIVA1 |  |  | 8654 | -0.529 | -0.3077 | Yes |
| 89 | ANP32B |  |  | 8680 | -0.545 | -0.2897 | Yes |
| 90 | H2AFV |  |  | 8693 | -0.554 | -0.2699 | Yes |
| 91 | TUBB |  |  | 8763 | -0.621 | -0.2540 | Yes |
| 92 | DEK |  |  | 8770 | -0.627 | -0.2307 | Yes |
| 93 | H2AFZ |  |  | 8774 | -0.630 | -0.2070 | Yes |
| 94 | STMN1 |  |  | 8783 | -0.638 | -0.1836 | Yes |
| 95 | LSM3 |  |  | 8803 | -0.655 | -0.1607 | Yes |
| 96 | RANBP1 |  |  | 8819 | -0.669 | -0.1369 | Yes |
| 97 | HIST1H4C |  |  | 8850 | -0.710 | -0.1132 | Yes |
| 98 | LSM4 |  |  | 8853 | -0.712 | -0.0863 | Yes |
| 99 | PTMA |  |  | 8894 | -0.764 | -0.0616 | Yes |
| 100 | UBE2S |  |  | 8901 | -0.772 | -0.0329 | Yes |
| 101 | TUBA1B |  |  | 8980 | -1.103 | 0.0005 | Yes |
Table: GSEA details [plain text format]

  

Fig 2: LARSSON\_STATE\_4: Random ES distribution      
 Gene set null distribution of ES for **LARSSON\_STATE\_4**
